# Provider preferences for postoperative analgesia in obese and non-obese patients undergoing ambulatory surgery

**DOI:** 10.1186/s40545-018-0138-x

**Published:** 2018-05-17

**Authors:** Anthony H. Bui, David L. Feldman, Michael L. Brodman, Peter Shamamian, Ronald N. Kaleya, Meg A. Rosenblatt, Debra D’Angelo, Donna Somerville, Santosh Mudiraj, Patricia Kischak, I. Michael Leitman

**Affiliations:** 1 Icahn School of Medicine at Mount Sinai, New York, NY 10029 USA; 2 Hospitals Insurance Company, New York, NY USA; 3 Montefiore Medical Center/Albert Einstein College of Medicine, Bronx, NY USA; 4 Maimonides Medical Center, Brooklyn, NY USA; 5 Department of Surgery, Icahn School of Medicine at Mount Sinai, One Gustave L. Levy Place, Box 1076, New York, NY 10029 USA

## Abstract

**Background:**

Few guidelines exist on safe prescription of postoperative analgesia to obese patients undergoing ambulatory surgery. This study examines the preferences of providers in the standard treatment of postoperative pain in the ambulatory setting.

**Methods:**

Providers from five academic medical centers within a single US city were surveyed from May–September 2015. They were asked to provide their preferred postoperative analgesic routine based upon the predicted severity of pain for obese and non-obese patients. McNemar’s tests for paired observations were performed to compare prescribing preferences for obese vs. non-obese patients. Fisher’s exact tests were performed to compare preferences based on experience: > 15 years vs. ≤15 years in practice, and attending vs. resident physicians.

**Results:**

A total of 452 providers responded out of a possible 695. For mild pain, 119 (26.4%) respondents prefer an opioid for obese patients vs. 140 (31.1%) for non-obese (*p* = 0.002); for moderate pain, 329 (72.7%) for obese patients vs. 348 (77.0%) for non-obese (*p* = 0.011); for severe pain, 398 (88.1%) for obese patients vs. 423 (93.6%) for non-obese (*p* < 0.001). Less experienced physicians are more likely to prefer an opioid for obese patients with moderate pain: 70 (62.0%) attending physicians with > 15 years in practice vs. 86 (74.5%) with ≤15 years (*p* = 0.047), and 177 (68.0%) attending physicians vs. 129 (83.0%) residents (*p* = 0.002).

**Conclusions:**

While there is a trend to prescribe less opioid analgesics to obese patients undergoing ambulatory surgery, these medications may still be over-prescribed. Less experienced physicians reported prescribing opioids to obese patients more frequently than more experienced physicians.

## Background

There is growing concern regarding overprescription of narcotic pain medication following ambulatory surgery. The use of postoperative opioid medication increases the risk of opioid-related morbidity, particularly gastrointestinal, cardiorespiratory, and central nervous system depression [1–3]. Furthermore, several studies have shown that prescribing opioid analgesia to patients following surgery may be associated with increased long-term use [4–7]. This is of particular concern given the increase in opioid use and opioid-related deaths over the last decade [8–11].

These risks are further magnified in obese patients undergoing surgery. Obese patients are at increased risk of opioid-induced respiratory complications [12–16]. Additionally, specific comorbidities associated with obesity - namely diabetes, heart failure, and pulmonary disease - have been shown to correlate with prolonged opioid use following major surgery [5]. In the United States, up to 80% of opioid-naive patients who underwent a low-risk surgical procedure from 2004 to 2012 filled a prescription for an opioid, and these rates increased over time [17]. Many of these prescriptions were found to be excessive or inappropriate [18, 19]. Several US studies have shown that patients often have leftover pills from their narcotic prescriptions following surgery, suggesting that such aggressive pain control protocols may be unnecessary [20, 21]. Regulations on prescription opioids are more stringent in Europe and other parts of the world, causing increased barriers to access to pain relief [22, 23]. However, while rates of opioid misuse are generally lower in Europe compared to the US, they have been increasing over the past few years [24].

The past decade has seen an increase in the prevalence of obesity and the number of ambulatory surgeries performed [25, 26]. However, postoperative pain management protocols are not well-described for obese patients in any field, bariatric surgery or otherwise. Despite the risks associated with postoperative opioid use and the evidence of over-prescription, few evidence-based academic or federal guidelines exist to date for the safe administration of opioid analgesia to obese patients following ambulatory surgery. A series of recommendations on postoperative pain management released by the American Pain Society does not specifically discuss special considerations for either obesity or ambulatory surgery [27]. Furthermore, the recent guidelines for prescribing opioids for chronic pain from the US Centers for Disease Control and Prevention makes only a brief mention of obesity, only to say that these patients are at increased risk of sleep apnea and therefore extra monitoring and careful titration should be used [28]. While there is a general recognition of the need to reduce the postoperative prescription of opioids to obese patients [14, 15, 29, 30], recommendations for alternative postoperative pain management methods have not translated into specific protocols for obese patients. Providers therefore face challenges in providing adequate pain control to obese patients while minimizing their risk of adverse events following surgery.

As such, there is a need to assess the attitudes and preferences of providers regarding their approach towards managing postoperative pain. This study surveys the preferences of providers in a large, urban network of academic health centers regarding their postoperative analgesic protocols of choice for their obese and non-obese patients following ambulatory surgery.

## Methods

### Setting

Providers caring for ambulatory surgery patients within a consortium of five medical centers in New York, NY from May through September 2015 were surveyed. These hospitals have a strong community presence and treat a diverse socioeconomic population with a wide adult age range. Providers surveyed were all of these hospitals with an appointment in a surgical department who have medication prescribing priveleges. While all analgesic orders are generally reviewed by the attending surgeon, all providers in this study have the ability to independently propose and place orders for pain medication. At each participating hospital in this study, ambulatory surgery is defined as any surgery in which the patient is discharged on the same day as the operation. The participating hospitals are insured by the same medical liability company directed by a group of physicians focused on patient safety. This company regularly undertakes patient safety evaluations and quality initiatives aimed at identifying and mitigating patient risk.

### Survey

For this pilot study, there was interest in evaluating the providers’ baseline attitudes regarding use of opioid analgesia in patients with obesity, a population known to be at increased risk for respiratory complications. Threfore, a structured questionnaire was developed with the aim of eliciting current practice habits from these providers. Survey participants were asked to indicate their role (surgical attending, surgical resident, physician assistant, or nurse practitioner), specialty, and years in practice (years as an attending for surgical attending physicians, post-graduate year for residents, post-training years for physician assistants or nurse practitioners). They were asked to indicate whether there is a BMI at which they will not perform ambulatory surgery on a patient, and if so, to indicate the BMI. They were then asked to provide their most frequently prescribed postoperative analgesic to obese and non-obese patients based upon the predicted severity of pain: mild, moderate, or severe. Survey participants either completed paper versions of the survey during departmental meetings or received an electronic version through e-mail. Responses were recorded into an encrypted database with uniquely generated, de-identified respondent IDs linked to further protect respondent identity as well as to prevent duplicate submissions.

### Primary analyses

The primary objective was to examine whether providers indicated different prescribing preferences for their obese vs. non-obese patients given the predicted severity of pain. Therefore, for each analgesic protocol indicated in the survey, the percent of providers that preferred the analgesic was calculated for both obese and non-obese patients for each pain severity level (mild, moderate, and severe). McNemar’s tests for paired observations were performed to compare the prescribing preferences of the providers for their obese vs. non-obese patients. For each pain severity level, the proportion of providers who indicated an opioid vs. a non-opioid protocol, as well as the proportion indicating each individual analgesic, were compared between obese and non-obese patients.

### Secondary analyses

The secondary objective was to examine the relationship between provider experience and prescribing preferences. First, attending physician respondents were categorized by years in practice, with more experienced attending physicians defined as those with greater than 15 years in practice, and less experienced defined as 15 years or fewer. This designation was a pragmatic decision based on surveys routinely distributed throughout the medical community that group providers similarly [31–33]. The percentage of more and less experienced attending physicians who preferred each analgesic protocol was calculated for both obese and non-obese patients for each pain severity level. Fisher’s exact tests were performed to compare the prescribing preferences of more vs. less experienced attending physicians. For each obesity status and pain severity level, the proportions of more vs. less experienced attending physicians who indicated an opioid vs. a non-opioid protocol, as well as the proportions indicating each individual analgesic, were compared. Second, the prescribing preferences of attending vs. resident physicians were examined. Fisher’s exact tests were performed to compare the prescribing preferences of attending and resident physicians similarly to the comparison of more vs. less experienced attending physicians. For all analyses, *p*-values less than 0.05 were considered significant.

## Results

### Study population

A total of 452 providers, out of a possible 695, responded to the survey for an overall response rate of 65%. Of these, 260 (57.5%) were surgical attending physicians, 155 (34.3%) were surgical residents, and 24 (5.3%) were physician assistants or nurse practitioners. Of the surgical attending physicians, 113 (43.5%) had been in practice for greater than 15 years, and 116 (44.6%) less than or equal to 15 years. Of the surgical residents, 34 (21.9%) were PGY-1, 35 (22.6%) PGY-2, 27 (17.4%) PGY-3, 30 (19.4%) PGY-4, 16 (10.3%) PGY-5, and 9 (5.8%) PGY-6 or greater. The most represented subspecialties were general surgery (130, 28.8%), obstetrics and gynecology (80, 17.7%), and orthopedic surgery (53, 11.7%) (Table 1). 145 (32.1%) respondents indicated that there is a BMI over which they would not perform ambulatory surgery on an obese patient; of these, 109 (75.2%) indicated this BMI to be > 40, 24 (16.6%) from 36 to 40, 6 (4.1%) from 30 to 35, and 2 (1.4%) < 30. The prevalence of obesity at the participating hospitals is 9–10%.

**Table 1:** Survey population: providers caring for ambulatory patients (*N* = 452)

	Number	Percent
Provider Type		
Surgical Attending	260	57.5
> 15 Years in Practice	113	43.5
< =15 Years in Practice	116	44.6
Not Reported	31	11.9
Surgical Resident	155	34.3
PGY-1	34	21.9
PGY-2	35	22.6
PGY-3	27	17.4
PGY-4	30	19.4
PGY-5	16	10.3
PGY-6 or Greater	9	5.8
Not Reported	4	2.6
Physician Assistant	22	4.9
Nurse Practitioner	2	0.4
Not Reported	13	2.9
Department/Specialty		
Department of Surgery		
General Surgery	130	28.8
Colorectal Surgery	15	3.3
Bariatric Surgery	13	2.9
Plastic Surgery	9	2
Vascular Surgery	9	2
Pediatric Surgery	3	0.7
Department of Obstetrics and Gynecology	80	17.7
Department of Orthopedic Surgery		
General Orthopedic Surgery	53	11.7
Podiatry	33	7.3
Department of Anesthesia	38	8.4
Department of Otolaryngology		
ENT/Head and Neck	25	5.5
Oral/Maxillofacial/Dental	3	0.7
Department of Urology	18	4
Department of Ophthalmology	16	3.5
Not Reported	7	1.5

### Prescribing preferences for obese vs. non-obese patients

Table 2 describes the preferred postoperative analgesics according to obesity status and pain level. Among providers who prescribe differently between obese and non-obese patients, there is a tendency to prescribe opioids less frequently to obese patients than to non-obese patients; this is consistent for all 3 pain severity levels.

**Table 2:** Provider preferences for postoperative pain management for obese vs. non-obese patients. results of McNemar’s paired tests

Obesity status	Obese, *n* (%)		Non-obese, *n* (%)		*p*-value
Pain Severity	Opioids	Non-opioids	Opioids	Non-opioids	
Mild pain	119 (26.4%)	333 (73.6%)	141 (31.1%)	311 (68.9%)	0.002
Moderate pain	329 (72.7%)	123 (27.3%)	348 (77.0%)	104 (23.0%)	0.011
Severe pain	398 (88.1%)	54 (11.9%)	423 (93.6%)	29 (6.4%)	< 0.001

For mild pain, 119 (26.4%) providers indicated an opioid as their analgesic of choice for obese patients compared to 141 (31.1%) providers for non-obese patients (*p* = 0.002). The most commonly listed analgesics for mild pain were acetaminophen, NSAIDs, and acetaminophen plus oxycodone. For moderate pain, 329 (72.7%) providers would prescribe an opioid to obese patients compared to 348 (77.0%) providers for non-obese patients (*p* = 0.011). For severe pain, 398 (88.1%) providers preferred an opioid for obese patients compared to 423 (93.6%) providers for non-obese patients (*p* < 0.001). For both moderate and severe pain, the most commonly preferred analgesics were acetaminophen plus oxycodone, acetaminophen plus codeine, and NSAIDs.

### Prescribing preferences by physician experience

Table 3 compares the preferred postoperative analgesics according to obesity status and pain level between more experienced attending physicians (> 15 years of experience) and less experienced ones (<=15 years). 70 (62%) more experienced attending physicians indicated an opioid as their choice analgesic for obese patients with moderate pain compared to 86 (74.5%) less experienced ones (*p* = 0.047). There were no significant differences between the proportions of more vs. less experienced attending physicians who indicated an opioid as their analgesic of choice for obese patients with either mild or severe pain.

**Table 3:** Physician preferences for postoperative pain management for obese vs. non-obese patients by experience and training. Results of fisher’s exact tests

Pain severity	Physician characteristics	Obese, *n* (%)		*p*-value	Non-Obese, *n* (%)		*p*-value
		Opioid	Non-Opioid		Opioid	Non-Opioid	
Mild Pain	Experience as Attending			0.767			1
	> 15 years	29 (26.0%)	84 (74.0%)		36 (32.0%)	77 (68.0%)	
	≤15 years	33 (28.6%)	83 (71.4%)		37 (31.8%)	79 (68.2%)	
	Physician Status			0.799			0.713
	Attending	68 (26.2%)	192 (73.8%)		79 (30.2%)	181 (69.8%)	
	Resident	43 (27.5%)	112 (72.5%)		50 (32.2%)	105 (67.8%)	
Moderate Pain	Experience as Attending			**0.047**			**0.023**
	> 15 years	70 (62.0%)	43 (38.0%)		75 (66.7%)	38 (33.3%)	
	≤15 years	86 (74.4%)	30 (25.6%)		93 (80.2%)	23 (19.8%)	
	Physician Status			**0.002**			**< 0.001**
	Attending	177 (68.0%)	83 (32.0%)		186 (71.7%)	74 (28.3%)	
	Resident	129 (83.0%)	26 (17.0%)		135 (87.1%)	20 (12.9%)	
Severe Pain	Experience as Attending			0.3156			0.106
	> 15 years	96 (84.9%)	17 (15.1%)		102 (90.0%)	11 (10.0%)	
	≤15 years	104 (89.5%)	12 (10.5%)		111 (96.0%)	5 (4.0%)	
	Physician Status			0.358			0.155
	Attending	230 (88.4%)	30 (11.6%)		241 (92.6%)	19 (7.4%)	
	Resident	142 (91.8%)	13 (8.2%)		150 (96.6%)	5 (3.4%)	

For non-obese patients with moderate pain, 75 (66.7%) more experienced attending physicians indicated an opioid as their choice analgesic compared to 93 (80.2%) less experienced attending physicians (*p* = 0.023). Similarly to obese patients, there were no significant differences between the proportions of more vs. less experienced physicians who indicated an opioid as their analgesic of choice for non-obese patients with either mild or severe pain.

Table 3 also compares the preferred postoperative analgesics according to obesity status and pain level between attending and resident physicians. 177 (68.0%) attending physicians indicated an opioid as their choice analgesic for obese patients with moderate pain compared to 129 (83.0%) residents (*p* = 0.002). There were no significant differences between the proportions of attending vs. resident physicians who indicated an opioid as their analgesic of choice for obese patients with either mild or severe pain.

For non-obese patients with moderate pain, 186 (71.7%) attending physicians indicated an opioid as their choice analgesic compared to 135 (87.1%) resident physicians (*p* < 0.001). Similar to obese patients, there were no significant differences between the proportions of attending vs. resident physicians who indicated an opioid as their analgesic of choice for non-obese patients with either mild or severe pain.

## Discussion

This study is one of the first to describe attitudes and preferences regarding opioid prescription to obese patients from the provider perspective. Most previous studies on this topic report data at the patient or the medical encounter level; i.e. how many patients received opioids, how many prescriptions for opioids were filled at pharmacies, or how many hospital visits included a prescription for opioids [34–37]. The prescribing preferences reported by providers in this study are consistent with a recognition of the unique risks of prescribing narcotic analgesia to postoperative obese patients. Fewer providers overall reported an opioid as their most frequently prescribed postoperative analgesic for their obese patients; this trend was consistent across all levels of pain severity. Of note, several providers in our study indicated tramadol as their analgesic of choice for obese patients for moderate pain. Tramadol is known to have a multi-modal mechanism of action including weak opioid receptor agonism and serotonin reuptake inhibition. It has demonstrated efficacy in treating moderate to severe postoperative pain and has been previously described as a potentially useful drug for patients who are at increased risk for respiratory complications, such as obese patients, due to its lower risk for respiratory depression compared to opioid analgesics [3, 38, 39].

However, the results from this study appear to confirm and contribute data toward the fact that providers may be overprescribing opioid analgesics to patients. Indeed, even for mild pain, about 30% of providers in this study stated their preference for an opioid analgesic. A notable result from this study was the finding that less experienced physicians – i.e., residents and attending physicians who have been in practice for 15 years or less – were more likely to prefer opioid medication for both their obese and non-obese patients with moderate pain compared to their more experienced counterparts. The results from this study may be suggestive of a difference in treatment priorities between younger and older physicians. It is possible that younger physicians are more concerned about preventing and treating their patients’ postoperative pain, whereas older physicians are more worried about opioid-related complications. Several reports from the primary care literature have found that younger physicians are less confident in their understanding of opioids, less confident with managing pain, and more reluctant to prescribe opioids, seemingly in conflict with the results of this study [40, 41]. However, one dermatology study found that younger dermatologists are more likely to prescribe opioids to their patients after Mohs surgery, which is consistent with the findings from this study [42]. The high outright rates of opioid prescription found in this study, and the discrepancy between younger and older physicians, both highlight opportunities for improved physician education on postoperative pain management and further demonstrates the need for safe analgesic guidelines.

Given the heterogeneity in postoperative pain management approaches found amongst providers in this study, and the possible gaps in understanding regarding pain control and opioid prescription that it highlights, there remains a need for standardized postoperative analgesic protocols. Recommendations for postoperative analgesia that are focused on obese patients emphasize the need to limit opioid use and propose strategies such as preoperative doses of NSAIDs, opioid alternatives, and multimodal analgesia [29, 30, 43–49]. However, these recommendations have not been fully synthesized into standard evidence-based protocols. Guidelines that do exist lack attention to the unique requirements of obese patients. For example, a series of recommendations for management of postoperative pain released jointly by the American Pain Society, the American Society of Regional Anesthesia and Pain Medicine, and the American Society of Anesthesiologists’ Committee on Regional Anesthesia includes strategies to reduce opioid requirements after surgery, but fails to discuss considerations specifically for obese patients [27]. As such, postoperative pain management continues to be driven by consensus rather than evidence, leading to variability in treatment and high rates of postoperative narcotic prescription to patients both with and without obesity.

This study has several limitations that are necessary to mention. First, all of the providers are employees of the participating hospitals within the consortium insured by the single medial liability company. All of these hospitals serve the New York City community. While this is a socioeconomically diverse community, the providers themselves may share common practices by virtue of being within a common academic setting. The results may not be generalizable to providers working in different settings, such as more rural communities or those in private/independent practices. Additionally, the survey relied on providers’ subjective interpretations of mild, moderate, and severe pain. One way to address this would have been to design a study in which clinical vignettes are given to the providers, and they are asked to propose an analgesic protocol. However, the primary objective of this study was to assess providers’ baseline attitudes and perceptions regarding postoperative analgesia for obese patients. It was felt that the more straightforward approach – i.e., asking directly about management with respect to pain severity – would avoid confounders such as age, demographics, and other comorbidities that, while allowing for a more standardized assessment of analgesic management, would detract from the primary question of pain control in obese vs. non-obese patients. Additionally, this was not meant to be an educational intervention, and there was concern that exposure to such vignettes would inadvertently lead to changes in patient care before a proper evaluation of baseline provider habits was assessed. Furthermore, providers were asked to identify a BMI above which they would not perform surgery. There is a risk that this oversimplifies the complexities involved in determining whether a patient is a candidate for surgery, including the patient’s comorbidities and the type of procedure being done. However, this study focused specifically on ambulatory surgery, which is defined in this study as surgery in which the patient leaves the hospital on the same day. This was felt to be a sufficiently narrow clinical scope for the purposes of this survey. Lastly, it is possible that the design of the survey could contribute towards survey bias. Providers were asked to fill in their preferred analgesic protocol for obese and non-obese patients for different levels of severity side-by-side. It is possible that providers would reflexively assume that they should input different responses upon seeing this format, when in practice their analgesic management of obese vs. non-obese management is not different. However, the fact that there was such a heterogeneous set of responses, including a high rate of providers who preferred opioids for mild pain as mentioned previously, seems to underline the initial motivation behind this study – that there are very few guidelines to aid in the management of these patients.

## Conclusion

This study, which aimed to survey of the prescribing preferences of providers for the pain management in obese and non-obese patients after ambulatory surgery, found that while providers tend to favor opioid analgesics less for their obese patients, many providers still prefer opioid analgesics even in situations when they may not be necessary. Furthermore. less experienced physicians may be prescribing narcotics at higher rates to both obese and non-obese patients than more experienced physicians. These results emphasize the need to develop post-discharge analgesic protocols with specific consideration for patients with obesity in order to provide adequate pain control while minimizing the risk of opioid-related adverse events.
